# Systemic LPS Translocation Activates Cross-Presenting Dendritic Cells but Is Dispensable for the Breakdown of CD8^+^ T Cell Peripheral Tolerance in Irradiated Mice

**DOI:** 10.1371/journal.pone.0130041

**Published:** 2015-06-15

**Authors:** Gabriel Espinosa-Carrasco, Marine Villard, Cecile Le Saout, Pascale Louis-Plence, Rita Vicente, Javier Hernandez

**Affiliations:** 1 Inserm U1183, Institute for Regenerative Medicine and Biotherapy, Montpellier, F-34295, France; 2 Université Montpellier, UFR de Médecine, Montpellier, F-34000, France; 3 CMRS/Laboratory of Immunoregulation, NIAID, NIH, Bethesda, Maryland, United States of America; Mie University Graduate School of Medicine, JAPAN

## Abstract

Lymphodepletion is currently used to enhance the efficacy of cytotoxic T lymphocyte adoptive transfer immunotherapy against cancer. This beneficial effect of conditioning regimens is due, at least in part, to promoting the breakdown of peripheral CD8^+^ T cell tolerance. Lymphodepletion by total body irradiation induces systemic translocation of commensal bacteria LPS from the gastrointestinal tract. Since LPS is a potent activator of the innate immune system, including antigen presenting dendritic cells, we hypothesized that LPS translocation could be required for the breakdown of peripheral tolerance observed in irradiated mice. To address this issue, we have treated irradiated mice with antibiotics in order to prevent LPS translocation and utilized them in T cell adoptive transfer experiments. Surprisingly, we found that despite of completely blocking LPS translocation into the bloodstream, antibiotic treatment did not prevent the breakdown of peripheral tolerance. Although irradiation induced the activation of cross-presenting CD8^+^ dendritic cells in the lymphoid tissue, LPS could not solely account for this effect. Activation of dendritic cells by mechanisms other than LPS translocation is sufficient to promote the differentiation of potentially autoreactive CD8^+^ T cells into effectors in irradiated mice. Our data indicate that LPS translocation is dispensable for the breakdown of CD8^+^ T cell tolerance in irradiated mice.

## Introduction

Microbiota of the gastrointestinal tract has multiple beneficial effects in the host, including the shaping of a fully functional immune system [1]. This symbiotic relationship is dependent on the ability of the host to keep bacteria secluded in the lumen. However, under certain pathological conditions the integrity of the gastrointestinal barrier may be compromised resulting in systemic microbial translocation (MT) [2]. Microbial products such as LPS, flagellin, peptidoglycan, unmethylated CpG rich DNA are potent activators of the innate immune system [3]. It is now well established that the inflammatory response to MT contributes to disease in inflammatory bowel disease patients as well as during HIV, HBV and HCV infection [4–6]. MT can also be triggered by immuno-depleting regimens used before hematopoietic stem cell transplantation. Since chemotherapeutic drugs and irradiation target rapidly dividing cells, they also affect the gastrointestinal barrier epithelial cells that have a fast turnover. In this scenario, MT-induced immune activation favors the onset of graft-versus-host disease where a major role for the LPS/TLR4 axis has been described [7–10].

T cell based immunotherapy is one of the most promising strategies against cancer [11, 12]. Conditioning regimens such as irradiation and chemotherapy are currently used in cancer patients because they critically enhance the efficacy of anti-tumor T cell adoptive transfer [13, 14]. At least three mechanisms have been evoked to explain this beneficial effect. First, lymphodepletion induced by conditioning protocols may result in a decrease in the number or functionality of suppressive cell populations [15]. Second, the hallmark of lymphopenia is the expansion and activation of residual or transferred T cells. Indeed, under acute lymphopenic conditions, naïve T cells proliferate in response to an increased availability of homeostatic cues, the cytokine IL-7 and weak TCR interactions with self-peptide/MHC complexes [16–21]. Importantly, lymphopenia induced proliferation (LIP) of naïve T cells is accompanied by a direct differentiation into cells that are functionally and phenotypically similar to memory cells, termed memory-like T cells, in the apparent absence of antigenic stimulation. Memory cells have a lower activation threshold. Therefore, expansion and activation of anti-tumor T cells would favor tumor rejection [22–26]. Third, as mentioned above, total body irradiation and chemotherapy induce MT [27]. LPS translocation after irradiation is responsible for the activation of antigen presenting dendritic cells (DC) via TLR4 signaling, which in turn are able to efficiently activate CD8^+^ T cells and enhance tumor rejection [27].

Many tumor-associated antigens are normal proteins expressed also in healthy tissues. Thus, efficient anti-tumor T cell based immunotherapeutic strategies would need to overcome the mechanisms of peripheral tolerance that prevent self-reactivity [28]. Interestingly, total body irradiation promotes the breakdown of self-tolerance and the development of efficient cytotoxic T lymphocyte (CTL) responses [29, 30]. Indeed, in a mouse model where the influenza virus hemagglutinin (HA) is expressed under the control of the rat insulin promoter in the beta cells of the pancreas, we have previously shown that HA-specific TCR transgenic CD8^+^ T cells undergo deletional tolerance, even in the presence of antigen-specific CD4^+^ T helper cells, upon self-antigen cross-presentation [31, 32]. Notably, the outcome was completely different when T cells were transferred into mildly irradiated hosts [29, 33]. Under these conditions, CD8^+^ T cells were able to overcome cross-tolerance and induce self-reactivity in a CD4^+^ T helper manner [33]. In irradiated mice, both HA-specific CD8^+^ and CD4^+^ T cells underwent extensive LIP and differentiated into memory-like cells. CD4^+^ T helper cells promoted the further differentiation of memory-like CD8^+^ T cells into effector CTL in response to antigen cross-presentation in the draining lymph nodes (LN) of the pancreas and their migration to the site of antigen-expression [33]. However, the mechanisms that promote the breakdown of peripheral cross-tolerance in irradiated mice remain unknown. In this study, in light of previous observations, we have addressed whether commensal bacteria LPS translocation is required to overcome CD8^+^ T cell self-tolerance in irradiated mice. We found that irradiation efficiently activated cross-presenting DC. However, LPS on its own could not account for full DC activation. Furthermore, LPS translocation was not required to overcome CD8^+^ T cell cross-tolerance. Differentiation of CD8^+^ T cells into effector CTL was equally efficient after LPS translocation blockade.

## Materials and Methods

### Ethics statement

Experimental procedures were conducted according to the European guidelines for animal welfare (2010/63/EU). Protocols were approved by the Animal Care and Use Committee “Languedoc-Roussillon” (approval number: CEEA-LR-12163). Blood withdrawal by intracardiac puncture was performed under isoflurane anesthesia. Diabetic mice were sacrificed to prevent suffering by prolonged hyperglycemia.

### Mice

BALB/c mice were purchased from Charles River and then housed at the Institut de Neuroscience de Montpellier (INM) animal facility. InsHA [34], Clone 4 TCR [35] and HNT TCR [36] transgenic mouse lines were backcrossed with BALB/c mice for at least 10 generations. Clone 4 and HNT mice were then crossed with BALB/c Thy1.1^+/+^ for two generations to achieve homozygosity for Thy1.1. Mice used in these studies were between 8 and 16 weeks of age. Mice were propagated and maintained under specific pathogen-free conditions at the INM animal facility.

### *In vivo* treatment with antibiotics and LPS

BALB/c or InsHA mice were treated with an antibiotic cocktail containing 1 g/L ampicillin, 1 g/L neomycin, 1 g/L metronidazole and 0.5 g/L vancomycin (Sigma-Aldrich) [37, 38] in the drinking water with bottle changes every 4–5 days. For DC phenotyping and LBP or cytokine measurement experiments mice were treated for 9 days until sacrifice, unless otherwise indicated. For adoptive transfer experiments, mice were treated for 13 days. Control BALB/c or InsHA mice were given regular drinking water.

BALB/c mice received 24h before sacrifice a single dose of 70μg of Ultrapure LPS from *E*. *coli* (Invitrogen) in PBS by i.p. injection.

### Mice irradiation

BALB/c or InsHA mice were sublethally irradiated (4.5 Gy) utilizing a therapeutic irradiator (Varian) 8 days after starting antibiotic treatment. Under these conditions, depletion of host T cells was approximately 80% at 48h after irradiation. Mice were then used for adoptive transfer experiments or for phenotypic analyses 24 h after irradiation.

### T cell isolation and adoptive transfer

Naïve CD8^**+**^ T cells from Clone 4 TCR Thy1.1 transgenic mice and CD4^+^ T cells from HNT Thy1.1 were prepared from LN and spleen by magnetic depletion using the T CD8^+^ and T CD4^+^ negative isolation kits (Dynabeads, Invitrogen) according to the manufacturer’s instructions. T cell purity was greater than 85%. Purified Clone 4 CD8^+^ T cells display a homogeneous naïve phenotype [29]. Isolated T cells (2x10^7^ cells/ml) were labeled with 2 μM of 5- and 6-carboxy-fluorescein succinimidyl ester (CFSE) (CellTrace CFSE Cell Proliferation Kit, Invitrogen) in PBS for 10 min at 37°C. Labeled Clone 4 TCR Thy1.1 CD8^+^ T cells and HNT TCR Thy1.1 CD4^+^ T cells were injected in PBS by i.v. injection.

### LBP measurement

Blood was collected by intracardiac puncture and plasma separated by centrifugation at 1000 *g*, 4°C, for 10 min. Plasma was stored at −80°C until further use. LBP was measured with the murine LBP ELISA kit according to the manufacturer's instructions (HyCult Biotechnology B.V., Uden, The Netherlands).

### Flow cytometry

For T cell phenotyping, pancreas, pancreatic lymph nodes (pLN) and a mixture of inguinal, axillary, cervical, mandibular, popliteal and mesenteric LN were excised and processed separately to obtain single cell suspensions by mechanical disruption on Nitex filters in PBS containing 2% FCS 0.02% sodium azide at 4°C. After counting, all pLN cells and an equivalent number of cells from other LN were stained with the indicated Abs. Cell suspensions from the pancreas were further subjected to ficoll (Histopaque) separation and all cells obtained from a single pancreas were stained. For DC phenotyping, all LN from single mice were pooled and digested in PBS containing 1 mg/ml Collagenase D (Roche) and 40 U/ml DNase I (Sigma-Aldrich) for 15 minutes at 37°C. After counting, equivalent numbers of cells from LN were stained with the indicated Abs.

The mAbs utilized were: anti-CD3-BV421, anti-CD3-APC, anti-CD19-BV421, anti-CD19-PerCPCy5.5, anti-DX5-BV421, anti-DX5-PE, anti-CD4-BV711, anti-CD4-FITC, anti-CD8a-BV786, anti-CD8a-Fitc, anti-CD11b-PE-CF594, anti-CD103-FITC, anti-Thy1.1-PerCP and anti-CD86-BV605 (BD PharMingen, San Diego CA); anti-CD11c-PE-Cy7, anti-CD25-APC-eFluor780, anti-GranzymB-PE, anti-F4/80-PE, anti-CD40-PerCP-eFluor710 and anti-MHC II (I-A/I-E)-AlexaFluor700 (eBioscience, San Diego, CA); anti-CD80-BV650 (BioLegend). After Fc-blockade, staining was performed in PBS containing 2% FCS and 0.02% sodium azide for 30 min at 4°C. Cells were then washed and analyzed on a FACSCanto II or a LSR Fortessa apparatus using Diva software (BDB, Mountain View, CA). Isotype-matched labeled antibodies were used as controls for specific staining.

Different cell populations were detected and enumerated by virtue of characteristic cell surface markers expression. For Clone 4 CD8^+^ T cells, the intensity of CFSE fluorescence was analyzed in the CD8^+^ Thy1.1^+^ gate. Intracellular Granzyme B staining was performed utilizing the Fixation and Permeabilization Kit (eBioscience) according to manufacturer’s instructions.

### Blood glucose monitoring

Mice were monitored for self-reactivity, induction of experimental diabetes, by measuring blood glucose every 3 days for a maximum period of 30 days after T cell transfer with a glucometer Breeze 2 Apparatus (Bayer, France). Animals were considered diabetic when glucose levels were above 300 mg/dl during two consecutive measurements.

### Statistical analyses

Statistical significance was determined using a Mann Whitney test with a one-tailed distribution and two-sample equal variance. Data were considered to be statistically different (*) for *P*<0.05, (**) for *P*<0.01, (***) for *P*<0.001.

## Results

### Irradiation induces LPS translocation and activation of multiple DC populations in BALB/c mice

**Fig 1:**
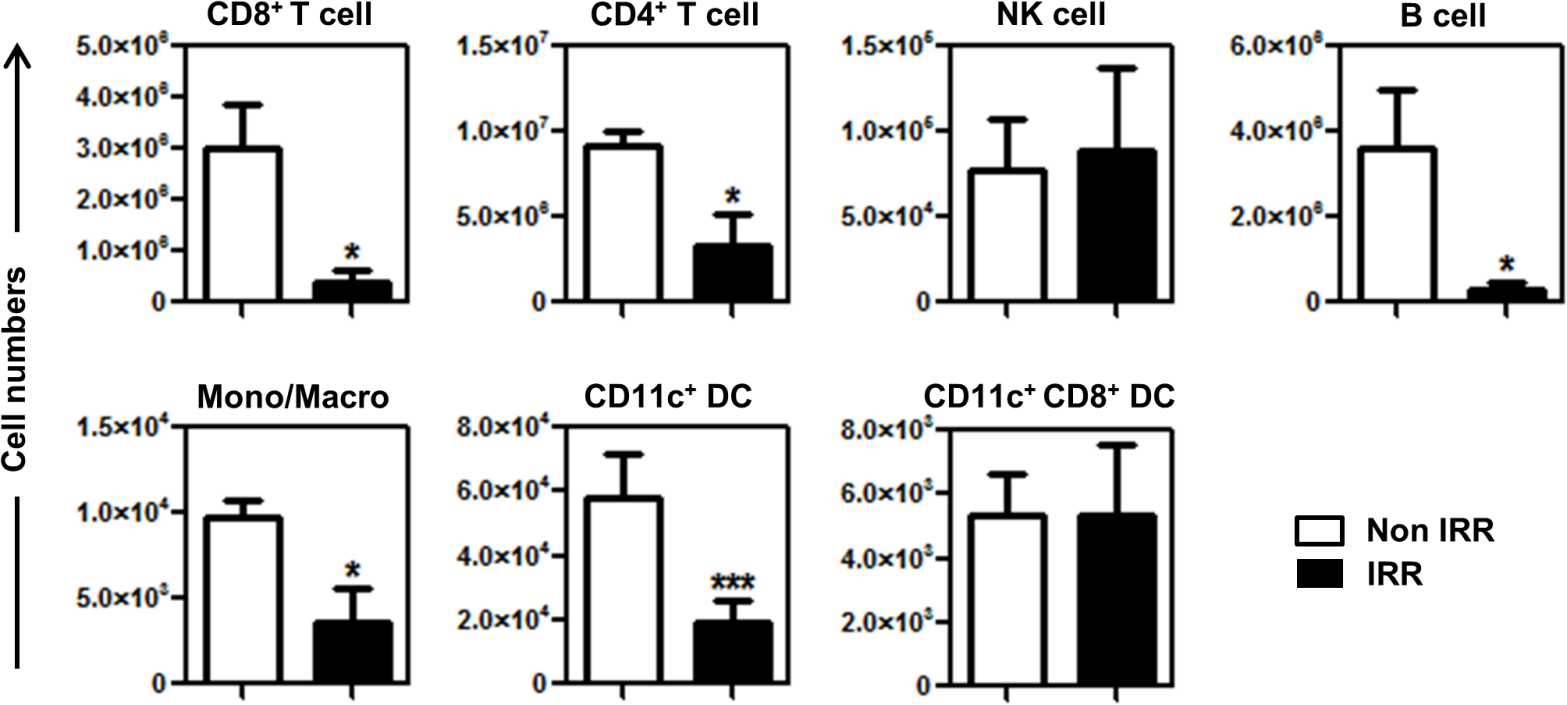
Depletion of immune cell populations in irradiated BALB/c mice. BALB/c mice were sacrificed 24h after irradiation, non-irradiated mice served as controls, and T cells, NK cells, B cells, Monocyte/Macrophages and DCs were enumerated. Absolute numbers of CD3^+^ CD8^+^ T cells, CD3^+^ CD4^+^ T cells, CD3^-^ DX5^+^ NK cells, CD19^+^ B cells, CD11b^+^ F4/80^+^ Mono/Macro, CD3^-^ CD19^-^ DX5^-^ CD11c^+^ MHC II^+^ DC and CD3^-^ CD19^-^ DX5^-^ CD11c^+^ MHC II^+^ CD8^+^ DC in the LN are represented as means ± SD (n = 4–8) from two independent experiments out of four.

**Fig 2:**
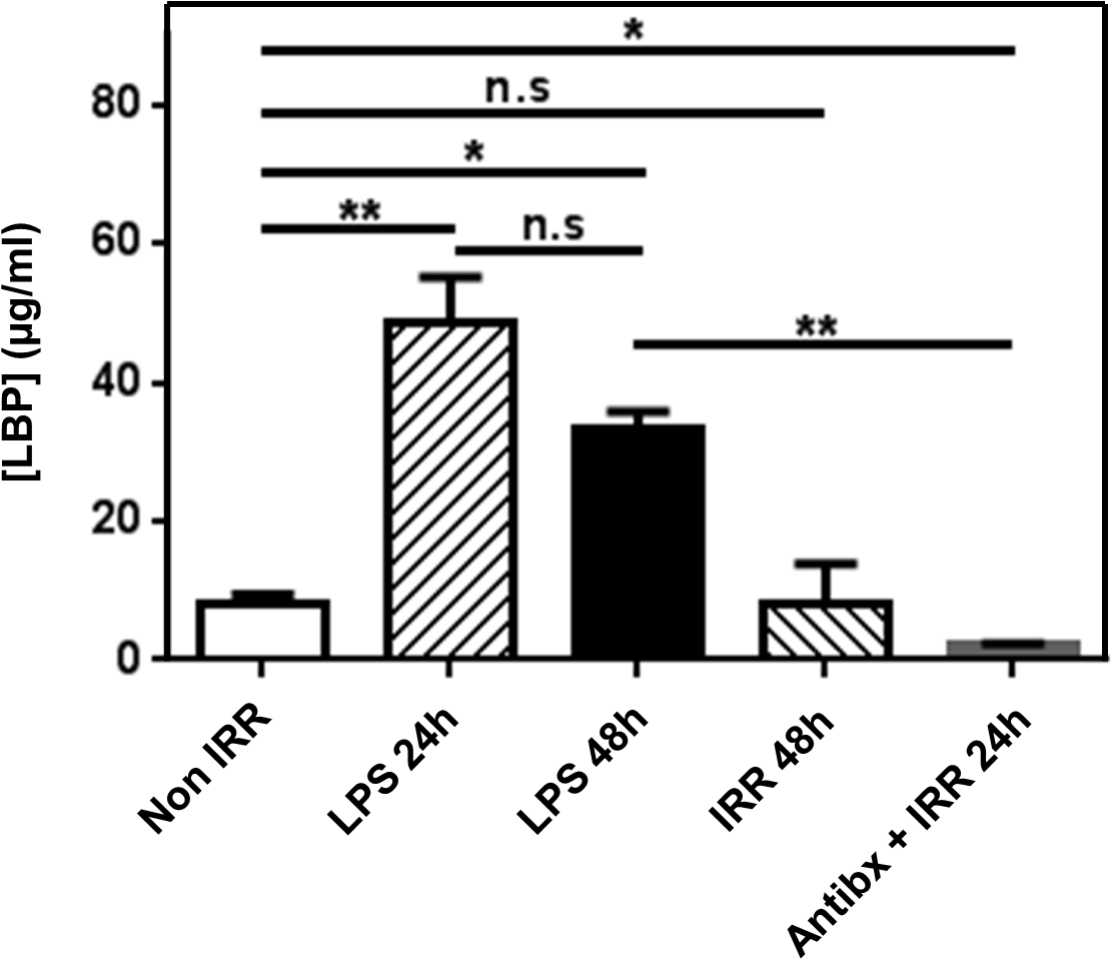
Irradiation-induced systemic LPS translocation is prevented by antibiotics in BALB/c mice. Sera from BALB/c mice were collected 24h (IRR 24h) or 48h (IRR 48h) after irradiation. Antibiotic-treated BALB/c mice were irradiated 8 days later and sera collected 24h after irradiation (Antibx + IRR 24h). Sera collected 24h after Ultrapure LPS i.p. injection from non-irradiated BALB/c mice served as positive control (LPS 24h). Sera from non-irradiated mice served as negative control (Non IRR). Concentration of LBP in serum is presented as means ± SD (n = 4–7) from 3 independent experiments.

Sublethal total body irradiation induces partial depletion of immune cells, leukopenia, and MT, which in turn are thought to promote cytokine secretion and activation of the remaining immune cells [27,39]. In BALB/c mice, leukocyte populations were differentially depleted 24h after irradiation (4.5 Gy). While CD8^+^ T cells and B cells were affected the most, NK cell numbers remained almost unchanged in the LN (Fig 1). As previously reported for C57BL/6 background mice [27], we assessed whether sublethal total body irradiation of BALB/c mice results in MT and the systemic release of LPS. For this purpose, we measured the concentration of LPS-binding protein (LBP), which parallels that of LPS in serum [40]. We found a highly significant 4.2-fold increase in the levels of LBP in sera of irradiated mice as compared to non-irradiated mice 24 h after irradiation (Fig 2). These levels were close to those observed in non-irradiated syngeneic mice that had been injected with Ultrapure LPS (Fig 2). However, 48 h after irradiation, LBP levels had declined and were not different from those in non-irradiated mice (Fig 2). These results indicate that irradiation mediates a transient, systemic LPS translocation in BALB/c mice.

**Fig 3:**
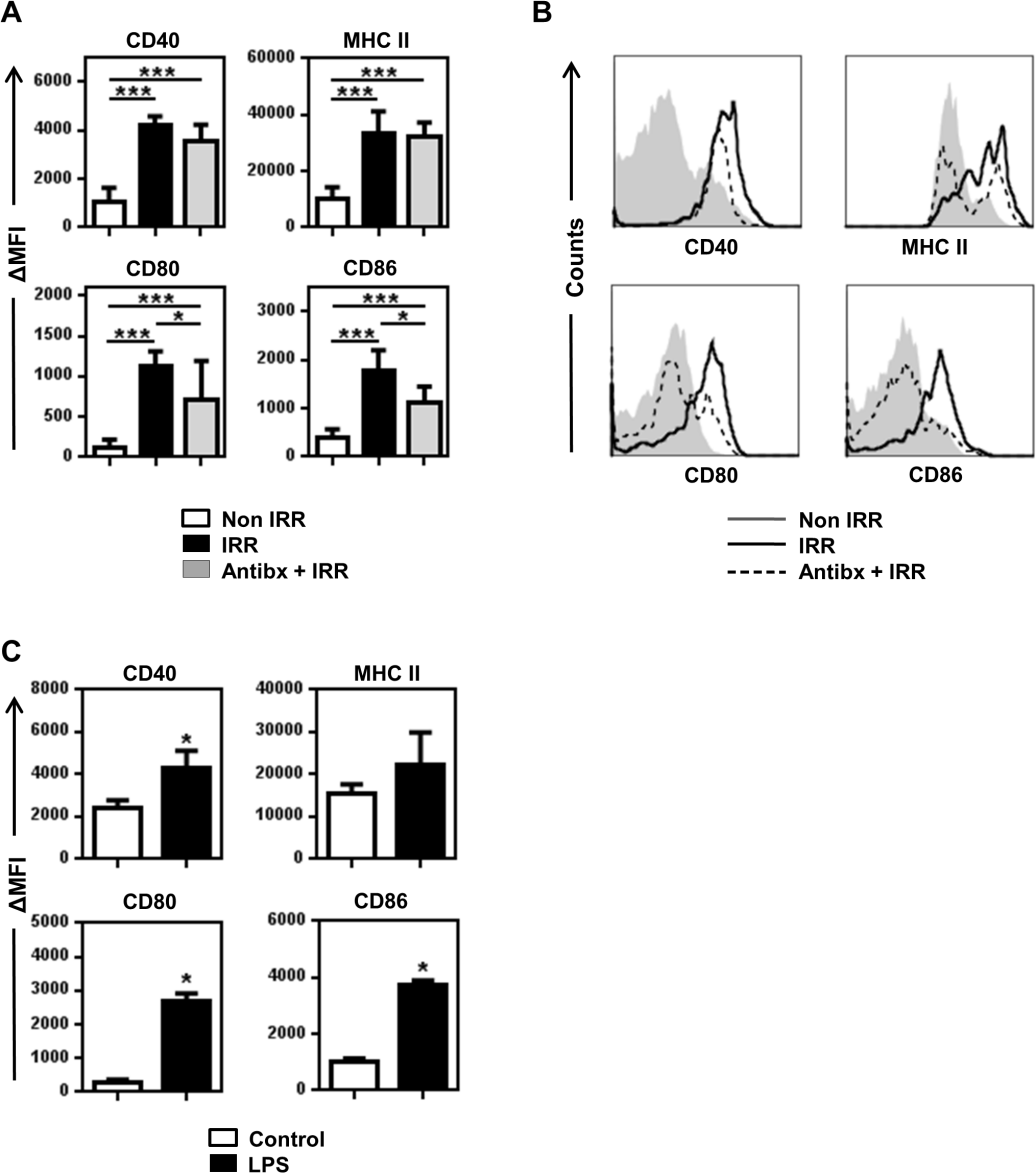
Antibiotics partially block irradiation-induced activation of CD11c^+^ DC. **(A)** Non-irradiated, irradiated and antibiotic-treated irradiated groups of BALB/c mice have been described in Fig 2. Mice were sacrificed 24h after irradiation and the expression of CD40, MHC class II, CD80 and CD86 on gated living CD3^-^ CD19^-^ DX5^-^ CD11c^+^ MHC II^+^ DC from the LN was analyzed by FACS. Increase in MFI respect to isotype-matched controls is represented as means ± SD (n = 6–8) from two independent experiments out of four. **(B)** Histograms represent the phenotype of CD3^-^ CD19^-^ DX5^-^ CD11c^+^ MHC II^+^ DC from individual representative mice described in panel A. **(C)** Non-irradiated and LPS-injected groups of mice described in Fig 2 were sacrificed 24 after treatment. Expression of CD40, MHC class II, CD80 and CD86 on gated living CD3^-^ CD19^-^ DX5^-^ CD11c^+^ MHC II^+^ DC was analyzed by FACS. Increase in MFI respect to isotype-matched controls is represented as means ± SD (n = 3–4) from two independent experiments out of three.

Irradiated mice presented increased levels of IL-12 and IL-6, but not of IL-1β, TNFα, IFNγ, IL-2 and IL-15, 24 h after irradiation (S1 Fig and data not shown). Furthermore, all the immune cell populations studied appeared to be in a more activated state than in non-irradiated mice, as evidenced by the high expression of CD69 or CD86 (S2 Fig). We focused on DCs as they are instrumental in the initiation of CTL responses. Irradiation also induced the activation of DCs in C57BL/6 mice, as indicated by the upregulation of CD86 in CD11c^+^ cells present in lymphoid organs [27]. We sought to extend this observation by analyzing a larger panel of activation markers, in well-defined DC populations, utilizing 13-parameter flow cytometry (see S3 Fig for gating strategy). Irradiation not only induced the upregulation of CD86 in total CD11^+^ DC (CD3^-^ CD19^-^ DX5^-^ CD11c^+^ MHC II^+^) in the LN but, also, strongly augmented the expression of CD80, CD40 and MHC class II molecules (Fig 3A and 3B). Next, we assessed the activation state of total DC in non-irradiated mice that had been injected with Ultrapure LPS. Interestingly, LPS induced the activation of CD11c^+^ DC in the lymph nodes as evidenced by the upregulation of CD80, CD86 and CD40 but it failed to induce a significant increase in the expression of MHC class II molecules (Fig 3C). Our data indicates that irradiation induces a strong activation of total DC in BALB/c mice that is not completely overlapping with that induced by LPS.

**Fig 4:**
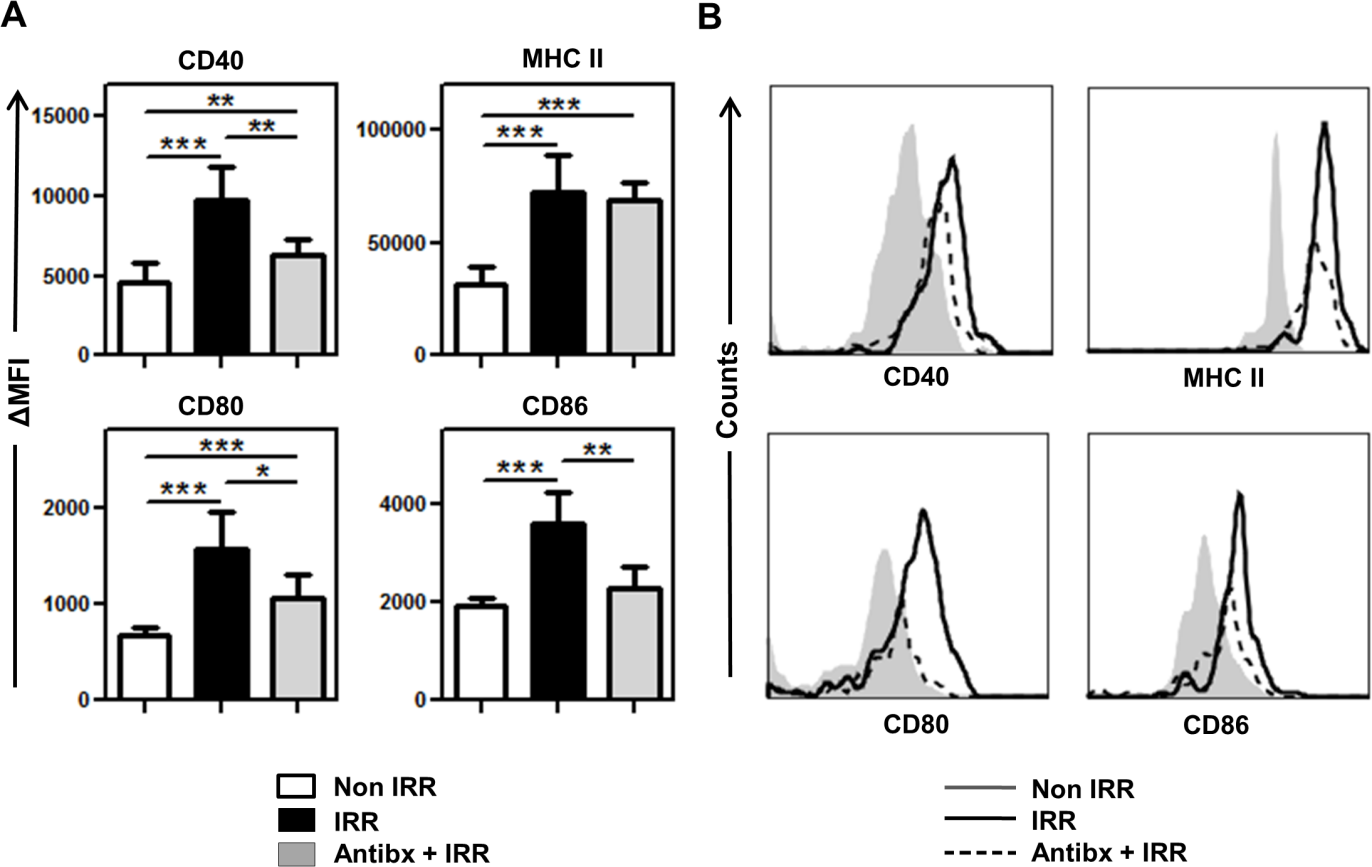
Antibiotics partially block irradiation-induced activation of CD8^+^ DC. **(A)** Non-irradiated, irradiated and antibiotic-treated irradiated groups of BALB/c mice have been described in Fig 2. Mice were sacrificed 24h after irradiation and the expression of CD40, MHC class II, CD80 and CD86 on gated living CD3^-^ CD19^-^ DX5^-^ CD11c^+^ MHC II^+^ CD8^+^ DC from the LN was analyzed by FACS. Increase in MFI respect to isotype-matched controls is represented as means ± SD (n = 6–8) from two independent experiments out of four. **(B)** Histograms represent the phenotype of CD3^-^ CD19^-^ DX5^-^ CD11c^+^ MHC II^+^ CD8^+^ DC from individual representative mice described in panel A.

DC cells are extremely heterogeneous and include populations with different functionality and origin [41]. Since our final interest is to assess the effect of irradiation in self-antigen cross-presentation, we assessed survival and activation of CD8^+^ DC (CD3^-^ CD19^-^ DX5^-^ CD11c^+^ MHC II^+^ CD8^+^, those mainly involved in self-antigen cross-presentation [42–44]) in the LN 24 h after irradiation (see S3 Fig for gating strategy). Irradiation induced a 3-fold decrease in the absolute numbers of total CD11c^+^ DC (Fig 1). However, CD8^+^ DC numbers remained unaffected after irradiation (Fig 1). Activation profiles of CD8^+^ DC were very similar to those of total DC, with a strong upregulation of all activation markers tested (Fig 4). Indeed, upregulation of CD80, CD86, CD40 and MHC class II was also observed in LN resident CD4^+^ DC (those that may be preferentially involved in stimulation of CD4+ T helper cells [45]) and migratory CD11b^+^ DC but not in migratory CD103^+^ DC, which only upregulated CD80 (S4 Fig). Taken together, these results show that the DC populations present in the LN are differentially affected by irradiation and that cross-presenting CD8^+^ DC are strongly activated in irradiated BALB/c mice.

### Antibiotic treatment prevents LPS translocation but only partially prevents DC activation

We focused our studies on LPS because in previous reports it could account for the whole effect of MT in enhancing anti-self tumor antigen CTL responses [9,10, 27]. To assess the role of systemic LPS translocation in the activation of DC, we have treated mice with a combination of different spectrum antibiotics (Cyprofloxacin, Neomycin, Ampicillin and Vancomycin) in the drinking water before irradiation [37, 38]. Antibiotics have been shown to prevent MT [27] and this particular cocktail to efficiently eliminate most of the gut commensal bacteria [37, 38]. We thought that utilizing a large spectrum antibiotic combination, rather than targeting gram negative bacteria exclusively, it would prevent the outgrowth of other microbial types that could occupy the available niche. This could imply innate activation through different patterns and receptors and mask the results obtained. In order to avoid the appearance of resistance associated with long-term treatments, we first assessed the optimal duration. We found that a treatment starting 8 days before irradiation completely prevented LPS translocation (Fig 2 and S5 Fig). Shorter (5 days) or longer (21 days) treatment periods resulted in LBP levels significantly higher than those in non-irradiated mice, most likely due to an incomplete bacterial clearance and the outgrowth of resistant bacteria respectively (S5 Fig). To confirm the absence of LPS translocation in antibiotic-treated mice, we assessed signaling through TLR4 in the liver. Translocated LPS from the gut first drain to the liver via the portal vein where is taken up by different cell types [46]. LPS uptake and stimulation of hepatocytes through TLR4 leads to upregulation of SOCS1 [47]. Therefore, we measured SOCS1 mRNA levels in the liver of irradiated mice as a read out of LPS/TLR4 signaling. We found a 4.7-fold increase in the expression of SOCS1 in irradiated mice compared to non-irradiated controls (S6 Fig). Notably, antibiotic-treatment during 8 days prior to irradiation completely prevented this increased expression induced by irradiation (S6 Fig). These results demonstrate that antibiotics prevent LPS translocation after irradiation.

Next, we assessed the phenotype of DC in the LN of antibiotic-treated mice after irradiation as compared to non-treated and non-irradiated groups. As previously reported [27], antibiotic treatment significantly diminished irradiation-induced upregulation of CD86 in total CD11c^+^ DC from LN (Fig 3A and 3B). However, we found varying effects in the expression of other markers tested. While antibiotic treatment partially prevented upregulation of CD80, it did not significantly modify the expression of CD40 and MHC class II that were strongly upregulated after irradiation (Fig 3A and 3C). We analyzed separately the phenotype of DC in the mesenteric LN, a place with an immediate access to translocated bacterial products, and found that, similarly to other LN, the strong activation induced by irradiation could not be completely reverted by antibiotics (S7 Fig). It is important to note that antibiotic treatment in non-irradiated mice had no effect on the expression of most of the markers under study (S8 Fig). The only noticeable effect was a decrease of CD86 levels compared to control mice (S8 Fig). We hypothesized that low level, constitutive MT [2] may be important to sustain basal levels of CD86 expression in DC. Antibiotic treatment might prevent this constitutive MT and interfere with CD86 basal expression. Indeed, LBP levels found in antibiotic-treated mice after irradiation are significantly lower than those in non-irradiated mice (Fig 2 and S5 Fig), suggesting that constitutive MT takes place in our model. Since LPS blockade could only partially prevent DC activation, we investigated whether cytokine secretion induced by irradiation followed the same pattern. Interestingly, antibiotic treatment completely prevented the accumulation of IL-12 and IL-6 in the sera of irradiated mice (S1 Fig). This supports the efficacy of our antibiotic treatment and indicates that contrary to DC activation, enhanced cytokine secretion is fully dependent of MT in irradiated mice.

Analysis of the resident CD8^+^ DC population in the LN of antibiotic-treated mice after irradiation revealed a significant reduction in the levels of CD80, CD86 and CD40 as compared to the same population in non-treated mice (Fig 4). However, the increase of MHC class II expression induced by irradiation was unaffected by the antibiotic treatment (Fig 4). As expected, the effect of antibiotics on the activation state of different DC varied depending on the population examined. Resident CD4^+^ DC behaved similarly to CD8^+^ DC in that antibiotic treatment significantly diminished the expression of CD80, CD86 and CD40 induced by irradiation but did not affect MCH class II levels (S4 Fig). In migratory CD11b^+^ DC, antibiotics prevented irradiation-induced activation almost completely as CD80, CD86 and CD40 levels were reduced to those found in non-irradiated mice and even MHC class II expression was strongly diminished (S4 Fig). On the other hand, antibiotics had little effect on the phenotype of CD103^+^ DC that were almost insensitive to activation by irradiation (S4 Fig). Taken together, our results show that antibiotics can completely prevent systemic LPS translocation induced by irradiation. However, DC activation was only partially reverted by this treatment, indicating that additional mechanisms differentially contribute to the activation of DC populations present in the LN.

### Antibiotic treatment does not prevent the breakdown of CD8^+^ T cell peripheral tolerance

**Fig 5:**
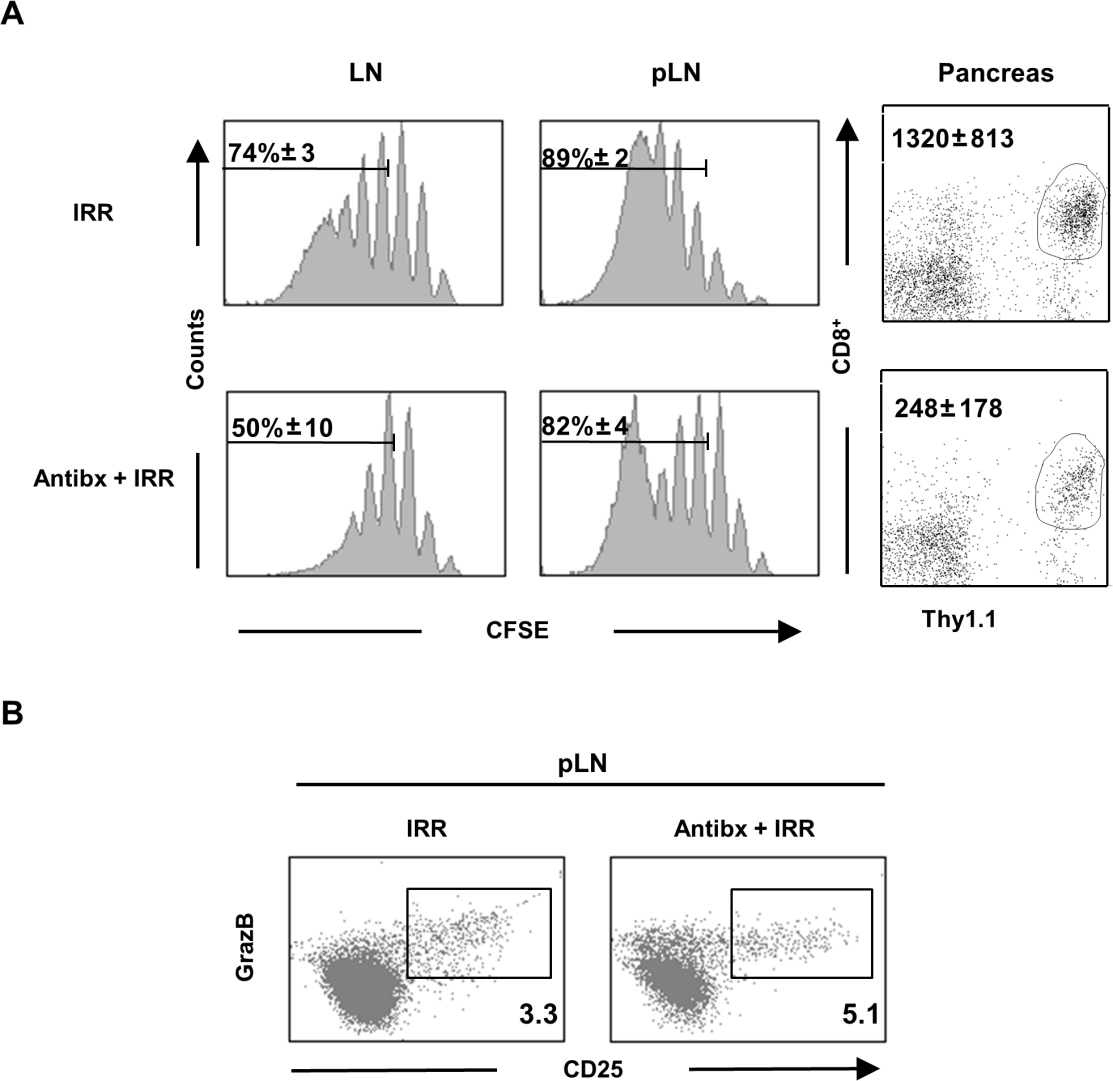
Antibiotics do not prevent antigen-driven proliferation and differentiation of Clone 4 CD8^+^ T cells into effectors in irradiated InsHA mice. **(A)** Irradiated and antibiotic-treated irradiated groups of InsHA mice adoptively transferred with 5x10^6^ CFSE-labeled naïve Clone 4 Thy1.1^+^ CD8^+^ and 5x10^6^ HNT CD4^+^ T cells were sacrificed on day 10 after transfer. CFSE fluorescence intensity on gated CD8^+^ Thy1.1^+^ donor lymphocytes in LN and pLN of single representative mice are shown in left and central panels. Percentages of highly proliferating cells, means ± SD (n = 4), of one experiment out three are presented. The presence of CD8^+^ Thy1.1^+^ donor T cells in the pancreas is shown in right panels. Numbers indicate FACS event counts in the depicted gates as means ± SD (n = 4) of one representative experiment out three. **(B)** Phenotype of CD8^+^ Thy1.1^+^ donor lymphocytes in the pLN of individual InsHA mice from groups described in panel A. Percentages of CD25^+^ Granzyme B^+^ of the donor T cells from pooled pLN of one experiment out of two.

Finally, we assessed whether antibiotic treatment is sufficient to prevent the breakdown of CD8^+^ T cell cross-tolerance as it occurs in irradiated mice. For this purpose, we utilized BALB/c InsHA mice where, under normal conditions, HA-specific Clone 4 TCR transgenic CD8^+^ T cells undergo deletional tolerance in the draining LN of the pancreas (pLN) mediated by antigen cross-presentation, even in the presence of HA-specific HNT TCR transgenic CD4^+^ T helper cells [31, 32]. On the other hand, when transferred into sublethally irradiated InsHA mice, Clone 4 CD8^+^ T cells undergo extensive LIP in non-draining LN and antigen-driven proliferation in the pLN ([29] and Fig 5A). Proliferating Clone 4 CD8^+^ T cells, with the help of cotransferred HNT CD4^+^ T cells, differentiate into effector CTL in the pLN and migrate to pancreas inducing self-reactivity ([29, 33], Fig 5 and Table 1). Antibiotic treatment in BALB/c InsHA mice was equally efficient in preventing LPS translocation and DC activation as in wild type BALB/c mice (data not shown). But, surprisingly, antibiotic treatment did not prevent self-reactivity in irradiated InsHA mice adoptively transferred with HA-specific CD8^+^ and CD4^+^ T cells, regardless of the dose of donor T cells utilized (Table 1). Analyses of the CFSE profiles of donor CD8^+^ T cells in non-draining LN showed that LIP was operative in antibiotic-treated mice, although it was moderately reduced when compared to non-treated mice (Fig 5A). An average of 50% of Clone 4 CD8^+^ T cells underwent more than 2 rounds of division in antibiotic treated mice versus 74% in non-treated controls (Fig 5A). Most importantly, self-antigen cross-presentation driven proliferation in the pLN was extensive and similar in both groups of mice (Fig 5A). Generation of effector CTL, as evidenced by the expression of CD25 and Granzyme B, was also not affected by antibiotics (Fig 5B) and effector CTL were able to migrate to the site of antigen expression, where self-reactivity ensued, in antibiotic-treated InsHA mice (Fig 5A). Thus, our results demonstrate that systemic LPS translocation is not required for the breakdown of CD8^+^ T cell peripheral tolerance and the generation of CTL in response to self-antigen cross-presentation in irradiated mice.

**Table 1:** Antibiotic treatment does not prevent self-reactivity in irradiated InsHA mice.

Treatment^a^	Cell transfer^b^	Diabetes incidence^c^
IRR	5x10^6^ + 5x10^6^	100% (n = 6) d9±2
Antibx + IRR		100% (n = 7) d10±4
IRR	1x10^6^ + 1x10^6^	80% (n = 5) d12±2
Antibx + IRR		100% (n = 6) d11±2

^a^ InsHA mice were treated with antibiotics or left untreated. 8 days later all mice were irradiated.

^b^ Equal numbers of purified transgenic Clone 4 CD8^+^ and HNT CD4^+^ T cells were injected as indicated into InsHA mice, 24h after irradiation.

^c^ The onset of self-reactivity was evaluated by measuring blood glucose levels. Mice were followed over a maximum of 30 days and were considered diabetic when levels were above 300mg/dl in two consecutive measurements.

## Discussion

Peripheral tolerance is essential to keep potentially autoreactive T cells under control and to prevent autoimmunity [48, 49]. However, since the tumor-associated antigens identified are often self-proteins, it also represents a barrier for the development of efficient anti-tumor responses [29]. The use of lymphodepleting regimens as adjuvants for adoptive transfer T cell immunotherapy against cancer represented a major advance for this strategy [11, 12]. Irradiation and chemotherapy dramatically enhanced its efficacy even when targeting self-tumor antigens [13, 14]. Lymphopenia is also a condition common to many animal models of autoimmunity and even present in patients with certain autoimmune diseases [50]. All these observations lead us and others to hypothesize that lymphodepletion perturbs the establishment of tolerance. Indeed, it has been recently shown that lymphodepletion promotes the breakdown of peripheral CD8^+^ T cell tolerance [29, 30]. In this report, we examined the role of systemic LPS translocation in the breakdown of CD8^+^ T cell cross-tolerance as it occurs in sublethally irradiated InsHA mice. Our results demonstrate that irradiation is a potent activator of cross-presenting CD8^+^ DC and that the other DC populations present in the LN respond differently to this stimulus. However, antibiotic treatment of mice prior to irradiation, which completely prevented LPS translocation and cytokine accumulation, could not prevent the breakdown of peripheral tolerance and the generation of effector CTL in response to self-antigen cross-presentation in the pLN. A likely explanation for these surprising results is that LPS translocation is not the only mechanism underlying cross-presenting DC activation induced by irradiation, as LPS translocation blockade could only partially prevent the activation of DC.

LPS has a major role in the onset of graft-versus-host disease after bone marrow transplantation where preparative myeloablative regimens induce MT [7, 8]. More recently, microbial LPS translocation and signaling through TLR4 have also been shown to account for the enhanced anti-self tumor antigen responses observed after irradiation and adoptive CD8^+^ T cell transfer in mouse models [27]. These observations suggest that LPS has a dominant role amongst the multiple microbial-associated patterns that during MT can induce innate activation to trigger efficient T cell responses. Our data, nonetheless, demonstrate that LPS does not have a major role in the breakdown of CD8^+^ T cell peripheral tolerance and in the generation of anti-self CTL responses after irradiation. A likely explanation for this apparent discrepancy between our results and those reported by Paulos et *al*. is the drastic difference in the experimental set up. While Paulos et *al*. utilized *in vitro* antigen-activated CD8^+^ T cells for adoptive transfer experiments [27], we have used naïve T cells. Non-activated CD8^+^ T cells encounter self-antigen cross-presented by DC in the LN. This is an important check-point in the establishment of peripheral tolerance *in vivo* [43]. Encounter with steady-state DC will induce tolerance. If DC are activated, CD8^+^ T cells will differentiate into effector CTL. Transfer of effector cells would bypass this critical check-point. Furthermore, although effector cells may also be subjected to tolerance, the mechanisms are likely to be rather different because they do not need co-stimulation for sustained function and expansion [49]. Our results demonstrate that the quality and magnitude of DC activation by translocated LPS differ from that induced by other factors provided by irradiation. While innate activation by LPS translocation appears to be sufficient to sustain effector cell activity against self-antigens in irradiated mice, it is neither sufficient nor required to transform a tolerogenic DC into a priming DC for non-activated CD8^+^ T cells.

Total body irradiation results in strong activation of most DC populations studied. However, our phenotypic analyses demonstrate that translocated LPS is only partly responsible for activation. MHC class II is, perhaps, the marker that better illustrates this conclusion. MHC class II is strongly upregulated after irradiation in both resident CD8^+^ and CD4^+^ DC. Nonetheless, LPS translocation blockade by antibiotic treatment has no effect on its expression in these two DC populations. On the other hand, injection of Ultrapure LPS does not significantly enhance MHC class II expression on DC *in vivo*. Moreover, previous reports have shown that injection of LPS does not efficiently provide priming signals for the generation of effector CTL *in vivo* [51]. What are, then, the other factors provided by irradiation promoting DC activation? Although in our studies we focused on LPS, a large spectrum bactericidal antibiotic cocktail was used to prevent MT. This cocktail has been previously described to eradicate all detectable bacteria from the gut of mice in experimental settings with a similar duration to ours [37, 38]. Therefore, it is unlikely that other microbial-associated patterns than LPS are responsible for activation. Irradiation, at the doses utilized here (4.5 Gy), induces massive death of hematopoietic cells. We found a 70% decrease in the cellularity of spleen and LN soon after irradiation. It is interesting to speculate that release of damage-associated molecular patterns (such as nucleic acids, ATP, uric acid HSPs and HMGB1) by dying cells may act as danger signals and contribute to the activation of DC after irradiation [52,53]. Indeed, it has been shown that HMGB1 released by dying tumor cells activates DC via TLR4 enhancing the efficacy of anti-tumor CTL responses [54]. Also, uric acid promotes DC activation, in an IL-1β dependent fashion, and contributes to the onset of graft-versus-host disease [55]. Additionally, since irradiation has multiple effects in the organism, other mechanisms than DC activation are likely involved in the breakdown of tolerance. LIP driven by increased IL-7 and the concomitant antigen-independent activation of CD8^+^ T cells have been shown to reverse anergy of CD8^+^ T cells [56]. Our results indicate that early release of proinflammatory cytokines, which is abolished by antibiotics, do not play a major role in the breakdown of tolerance. However, many different cell populations are still activated in antibiotic treated mice and would be able to produce cytokines at later time points after irradiation. Indeed, the increased availability of cytokines other than IL-7 observed in irradiated mice has been shown to contribute to the gain of effector functions by T cells [30, 57]. Finally, the removal of suppressive populations involved in maintaining T cells under control [15, 57]. Futures studies will help elucidate the relative contribution of these mechanisms.

An unexpected observation in the adoptive transfer experiments presented here is that antibiotics reduce the extent of LIP in irradiated mice. Suggesting that some of the cells undergoing LIP may be responding to commensal bacteria derived antigens. However, it has been clearly demonstrated that CD8^+^ T cell LIP in irradiated mice is due to homeostatic cues and not to foreign antigens [58]. Indeed, proliferation of Clone 4 CD8^+^ T cells in irradiated syngeneic mice can be completely abrogated by IL-7 signaling blockade (our unpublished observations). Interestingly, it has been recently shown that microbiota have an important role in regulating the production of IL-7 in the organism and germ-free mice have much lower IL-7 levels [59]. Therefore, antibiotic treatment may reduce the levels of available IL-7, which is critical for LIP, and reduce the extent of Clone 4 CD8^+^ T cell proliferation.

In conclusion, our results demonstrate that microbial LPS translocation is not required to overcome deletional tolerance induced by self-antigen cross-presentation in irradiated mice. Total body irradiation is a potent activator of multiple DC populations, including those thought to be involved in self-antigen cross-presentation to CD8^+^ T cells and those that may preferentially stimulate CD4^+^ T helper cells in the lymphoid tissue. However, LPS cannot account on its own for this effect. In irradiated mice where MT was blocked, CD8^+^ T cells are still able to become effector CTL due to additional cues provided by irradiation. These data have important implications for the understanding of autoimmune processes under lymphopenic conditions as well as for the development of T cell based cancer immunotherapies.

## Supporting Information

S1 Fig Antibiotics prevent irradiation-induced IL-12 and IL-6 production in irradiated BALB/c mice. Sera from BALB/c mice were collected 24h (IRR) after irradiation. Antibiotic-treated BALB/c mice were irradiated 8 days later and sera collected 24h after irradiation (Antibx + IRR). Sera collected 24h after Ultrapure LPS i.p. injection from non-irradiated Balb/c mice served as positive control (LPS). Sera from non-irradiate mice served as negative control (Non IRR). Cytokine protein concentration was determined by ELISA with the Mouse IL-12p70 DuoSet Kit **(A)** and the Mouse IL-6 DuoSet Kit **(B)** according to manufacturer’s instructions. Concentration in serum is presented as means ± SD (n = 4) from 2 independent experiments.
(TIF)

S2 Fig Irradiation induces activation of multiple immune cell populations. Non-irradiated, irradiated and antibiotic-treated irradiated groups of BALB/c mice have been described in Fig 2. Mice were sacrificed 24h after irradiation and the expression of CD69 on gated living CD3^+^ CD8^+^ T cells, CD3^+^ CD4^+^ T cells, CD3^-^ DX5^+^ NK cells as well as CD86 on gated living CD19^+^ B cells and CD11b^+^ F4/80^+^ Monocyte/Macrophages from the LN were analyzed by FACS. Increase in MFI respect to isotype-matched controls is represented as means ± SD (n = 3–4) from one representative experiment out of two.
(TIF)

S3 Fig Gating strategy for the analysis of DC populations in the LN. Single cell suspensions from collagenase-digested, pooled LN of individual BALB/c were stained and analyzed as described in Materials and Methods. FSC and SSC were used to exclude dead cells and doublets (upper left and central panels). T, NKT, B and NK cells were excluded by the use of CD3, CD19 and DX5 mAbs (upper right panel). Gate 1 represents total DC identified as CD3^-^ CD19^-^ DX5^-^ CD11c^+^ MHC II^+^ (lower left panel). Then, a combined gate of CD11c^hi^ and MHC II^hi^ cells was used for the analysis of conventional LN resident and migratory DC. Gate 2 represents resident CD8^+^ DC identified as CD3^-^ CD19^-^ DX5^-^ CD11c^+^ MHC II^+^ CD8^+^ (lower central panel). Gate 3 represents resident CD4^+^ DC identified as CD3^-^ CD19^-^ DX5^-^ CD11c^+^ MHC II^+^ CD4^+^ (lower central panel). Gate 4 represents migratory CD103^+^ DC identified as CD3^-^ CD19^-^ DX5^-^ CD11c^+^ MHC II^+^ CD8^-^ CD4^-^ CD103^+^ (lower right panel). And gate 5 represents migratory CD11b^+^ DC identified as CD3^-^ CD19^-^ DX5^-^ CD11c^+^ MHC II^+^ CD8^-^ CD4^-^ CD11b^+^ (lower right panel). Strategy was adapted from Helft et al. (Helft J, Manicassamy B, Guermonprez P, Hashimoto D, Silvin A, Agudo J, Brown BD, Schmolke M, Miller JC, Leboeuf M, Murphy KM, García-Sastre A, Merad M. Cross-presenting CD103^+^ dendritic cells are protected from influenza virus infection. J Clin Invest. 2012. 122:4037–47)
(TIF)

S4 Fig Antibiotics partially blocks activation of CD4^+^, CD11b^+^ and CD103^+^ DC after irradiation. Non-irradiated, Irradiated and Antibiotic-treated irradiated groups of BALB/c mice have been described in Fig 2. Mice were sacrificed 24h after irradiation and the expression of CD40, MHC class II, CD80 and CD86 on gated CD4+ DC **(A)**, CD11b+ DC **(B)** and CD103+ DC **(C)**, as defined in S1 Fig, from the LN was analyzed by FACS. Increase in MFI respect to isotype-matched controls is represented as means ± SD (n = 6–8) from two independent experiments out of three.
(TIF)

S5 Fig Optimal duration of antibiotic-treatment to prevent systemic LPS translocation in irradiated mice. BALB/c mice were treated with antibiotics for different lengths of time, as indicated, before irradiation. Sera were collected 24h after irradiation. Sera from non-irradiated and irradiated mice served as negative and positive controls respectively. Concentration of LBP in serum is presented as means ± SD (n = 4–6) and compared to non-irradiated mice for statistical significance.
(TIF)

S6 Fig Antibiotics prevent irradiation-induced SOCS1 expression in the liver. Liver samples from non-treated (Non IRR), irradiated (IRR) and antibiotic-treated irradiated (Antibx + IRR) BALB/c mice were collected 24 h after irradiation and immerged in 5 volumes of RNAlater solution (Ambion). Total RNA extraction was performed using QIAzol Lysis Reagent (Qiagen) and the RNA samples were treated with RQ1 RNase-Free DNase (Promega) to remove genomic DNA contamination. cDNA was synthetized from 500 ng of RNA using the high-capacity cDNA reverse transcription kit (Applied Biosystem). Real-time qPCR was performed using TaqMan specific primers (SOCS1 and Gapdh I.D. of Mm00782550_s1 and Mm99999915_g1 respectively) and TaqMan Universal PCR Master Mix (Applied Biosystem). SOCS1 mRNA relative expression levels are represented as mean ± SD (n = 3–5). Experiment was performed three times.
(TIF)

S7 Fig Antibiotics partially block irradiation-induced activation of CD11c^+^ DC in the mesenteric LN. Non-irradiated, irradiated and antibiotic-treated irradiated groups of BALB/c mice have been described in Fig 2. Mice were sacrificed 24h after irradiation and the expression of CD40, MHC class II, CD80 and CD86 on gated living CD3^-^ CD19^-^ DX5^-^ CD11c^+^ MHC II^+^ DC from the mesenteric LN was analyzed by FACS. Increase in MFI respect to isotype-matched controls is represented as means ± SD (n = 3–4) from one representative experiment out of three.
(TIF)

S8 Fig The effect of antibiotics on the phenotype of CD11c^+^ DC in the LN of non-irradiated mice. Antibiotic-treated BALB/c mice and non-treated controls were sacrificed 8 days after starting treatment. Expression of CD40, MHC class II, CD80 and CD86 on gated living CD3^-^ CD19^-^ DX5^-^ CD11c^+^ MHC II^+^ DC was analyzed by FACS. Increase in MFI respect to isotype-matched controls is represented as means ± SD (n = 3–4) from one out two independent experiments.
(TIF)
