# Supplementary figures and images for: Systemic LPS Translocation Activates Cross-Presenting Dendritic Cells but Is Dispensable for the Breakdown of CD8+ T Cell Peripheral Tolerance in Irradiated Mice
Source: PLoS One. 2015 Jun 15;10(6):e0130041. doi: 10.1371/journal.pone.0130041 (PMC4468093; doi:10.1371/journal.pone.0130041)

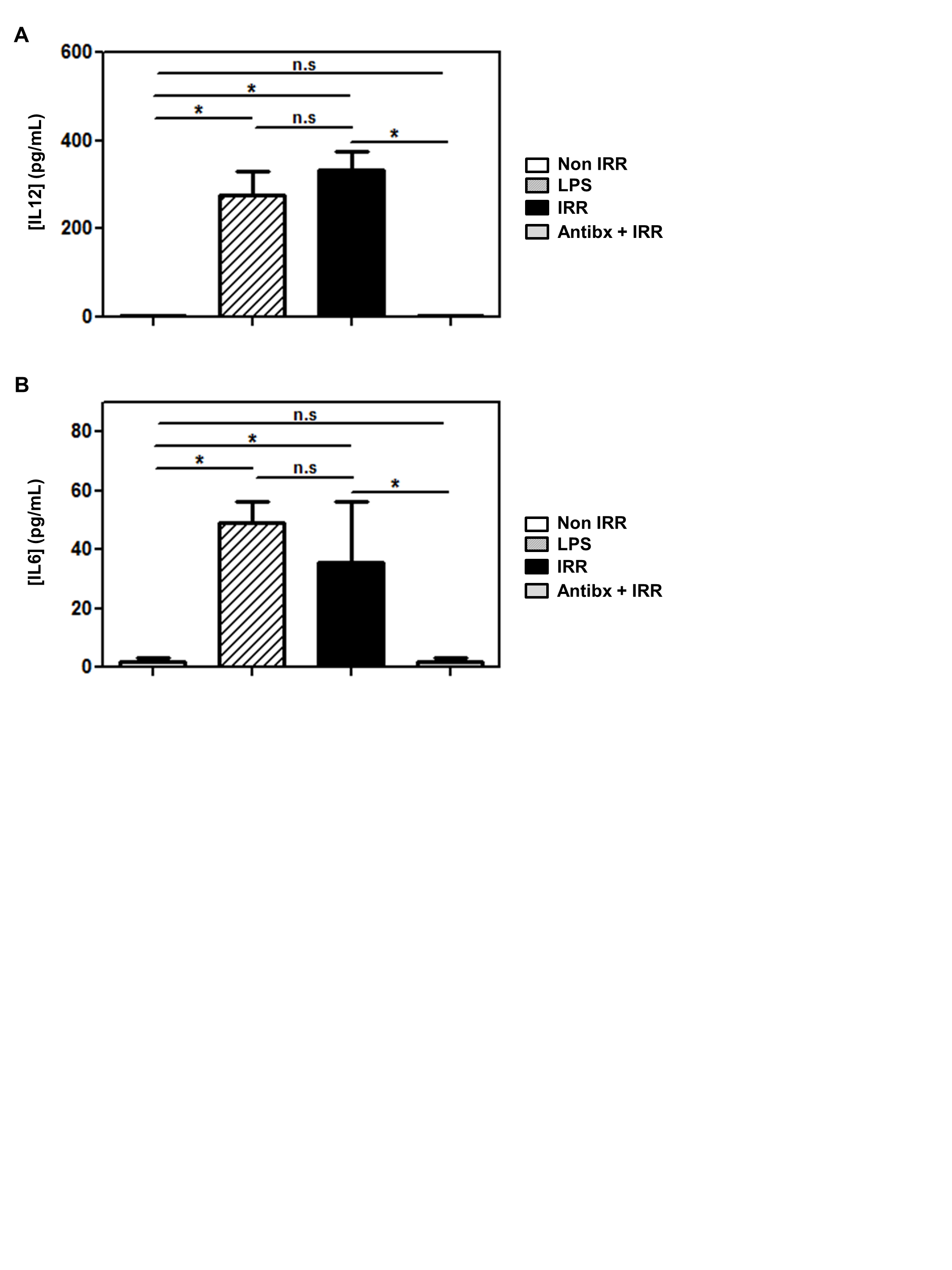

Supplement: S1 Fig — Sera from BALB/c mice were collected 24h (IRR) after irradiation. Antibiotic-treated BALB/c mice were irradiated 8 days later and sera collected 24h after irradiation (Antibx + IRR). Sera collected 24h after Ultrapure LPS i.p. injection from non-irradiated Balb/c mice served as positive control (LPS). Sera from non-irradiate mice served as negative control (Non IRR). Cytokine protein concentration was determined by ELISA with the Mouse IL-12p70 DuoSet Kit (A) and the Mouse IL-6 DuoSet Kit (B) according to manufacturer’s instructions. Concentration in serum is presented as means ± SD (n = 4) from 2 independent experiments. (TIF) [file pone.0130041.s001.tif]

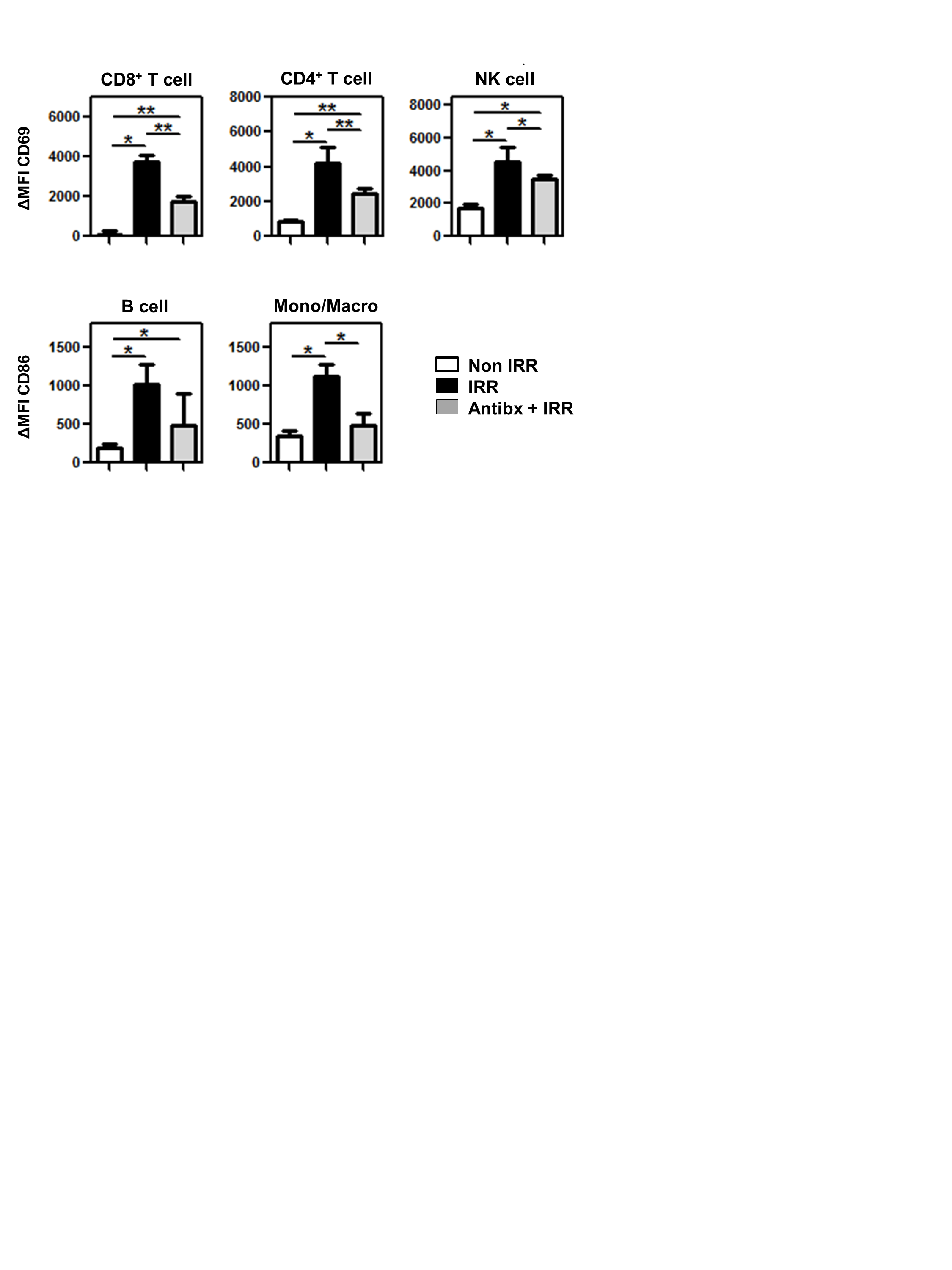

Supplement: S2 Fig — Non-irradiated, irradiated and antibiotic-treated irradiated groups of BALB/c mice have been described in Fig 2. Mice were sacrificed 24h after irradiation and the expression of CD69 on gated living CD3+ CD8+ T cells, CD3+ CD4+ T cells, CD3- DX5+ NK cells as well as CD86 on gated living CD19+ B cells and CD11b+ F4/80+ Monocyte/Macrophages from the LN were analyzed by FACS. Increase in MFI respect to isotype-matched controls is represented as means ± SD (n = 3–4) from one representative experiment out of two. (TIF) [file pone.0130041.s002.tif]

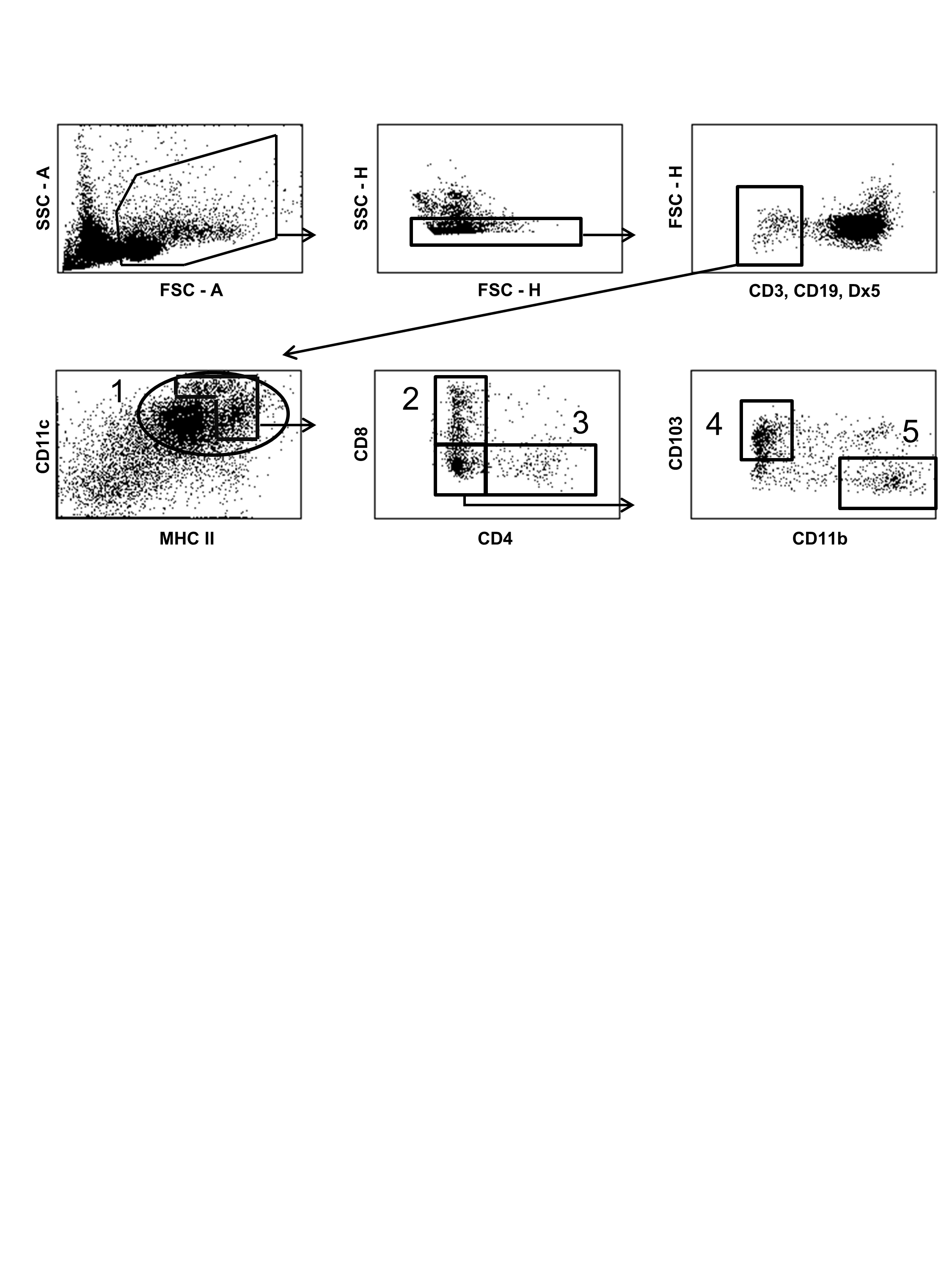

Supplement: S3 Fig — Single cell suspensions from collagenase-digested, pooled LN of individual BALB/c were stained and analyzed as described in Materials and Methods. FSC and SSC were used to exclude dead cells and doublets (upper left and central panels). T, NKT, B and NK cells were excluded by the use of CD3, CD19 and DX5 mAbs (upper right panel). Gate 1 represents total DC identified as CD3- CD19- DX5- CD11c+ MHC II+ (lower left panel). Then, a combined gate of CD11chi and MHC IIhi cells was used for the analysis of conventional LN resident and migratory DC. Gate 2 represents resident CD8+ DC identified as CD3- CD19- DX5- CD11c+ MHC II+ CD8+ (lower central panel). Gate 3 represents resident CD4+ DC identified as CD3- CD19- DX5- CD11c+ MHC II+ CD4+ (lower central panel). Gate 4 represents migratory CD103+ DC identified as CD3- CD19- DX5- CD11c+ MHC II+ CD8- CD4- CD103+ (lower right panel). And gate 5 represents migratory CD11b+ DC identified as CD3- CD19- DX5- CD11c+ MHC II+ CD8- CD4- CD11b+ (lower right panel). Strategy was adapted from Helft et al. (Helft J, Manicassamy B, Guermonprez P, Hashimoto D, Silvin A, Agudo J, Brown BD, Schmolke M, Miller JC, Leboeuf M, Murphy KM, García-Sastre A, Merad M. Cross-presenting CD103+ dendritic cells are protected from influenza virus infection. J Clin Invest. 2012. 122:4037–47) (TIF) [file pone.0130041.s003.tif]

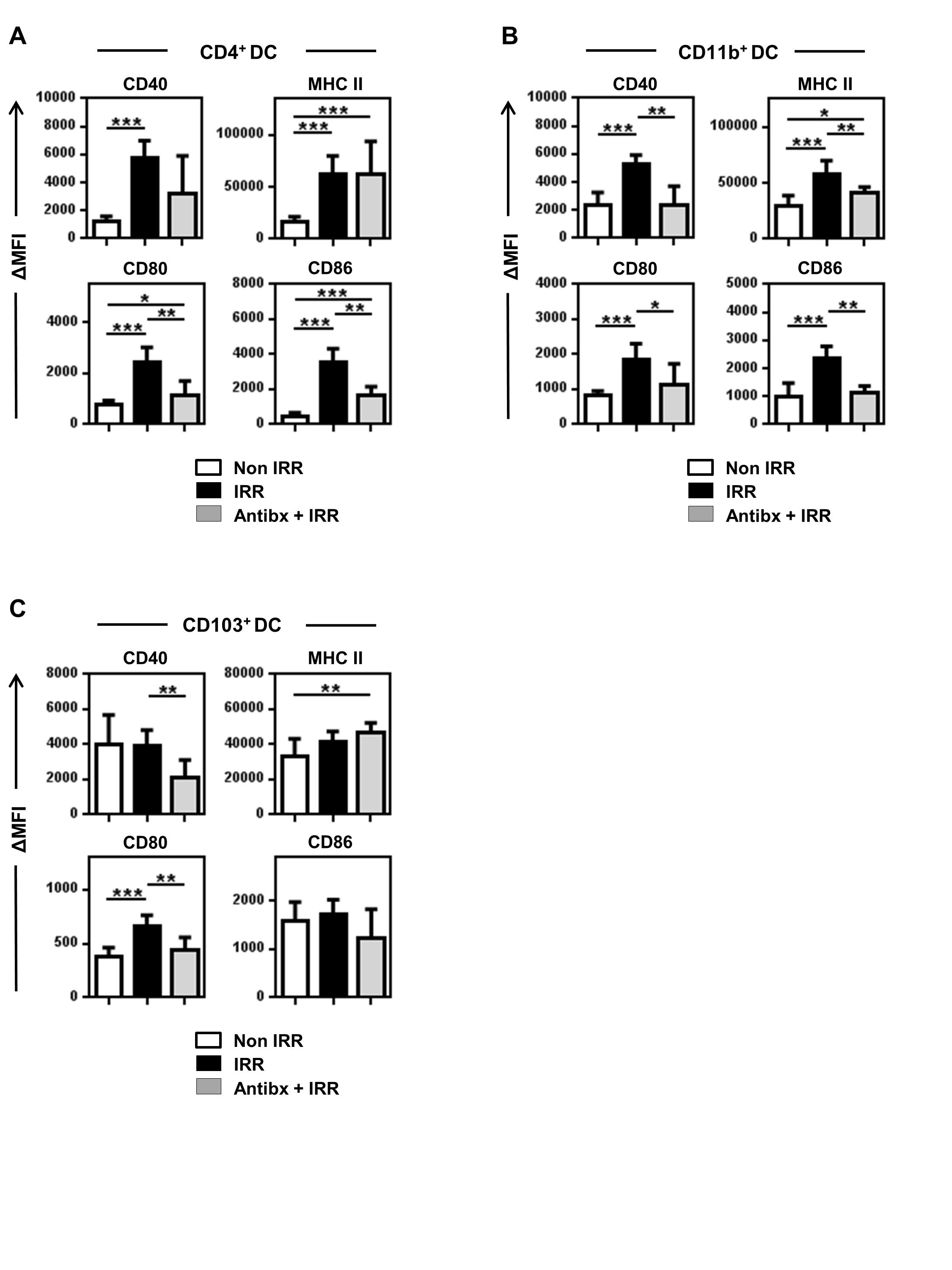

Supplement: S4 Fig — Non-irradiated, Irradiated and Antibiotic-treated irradiated groups of BALB/c mice have been described in Fig 2. Mice were sacrificed 24h after irradiation and the expression of CD40, MHC class II, CD80 and CD86 on gated CD4+ DC (A), CD11b+ DC (B) and CD103+ DC (C), as defined in S1 Fig, from the LN was analyzed by FACS. Increase in MFI respect to isotype-matched controls is represented as means ± SD (n = 6–8) from two independent experiments out of three. (TIF) [file pone.0130041.s004.tif]

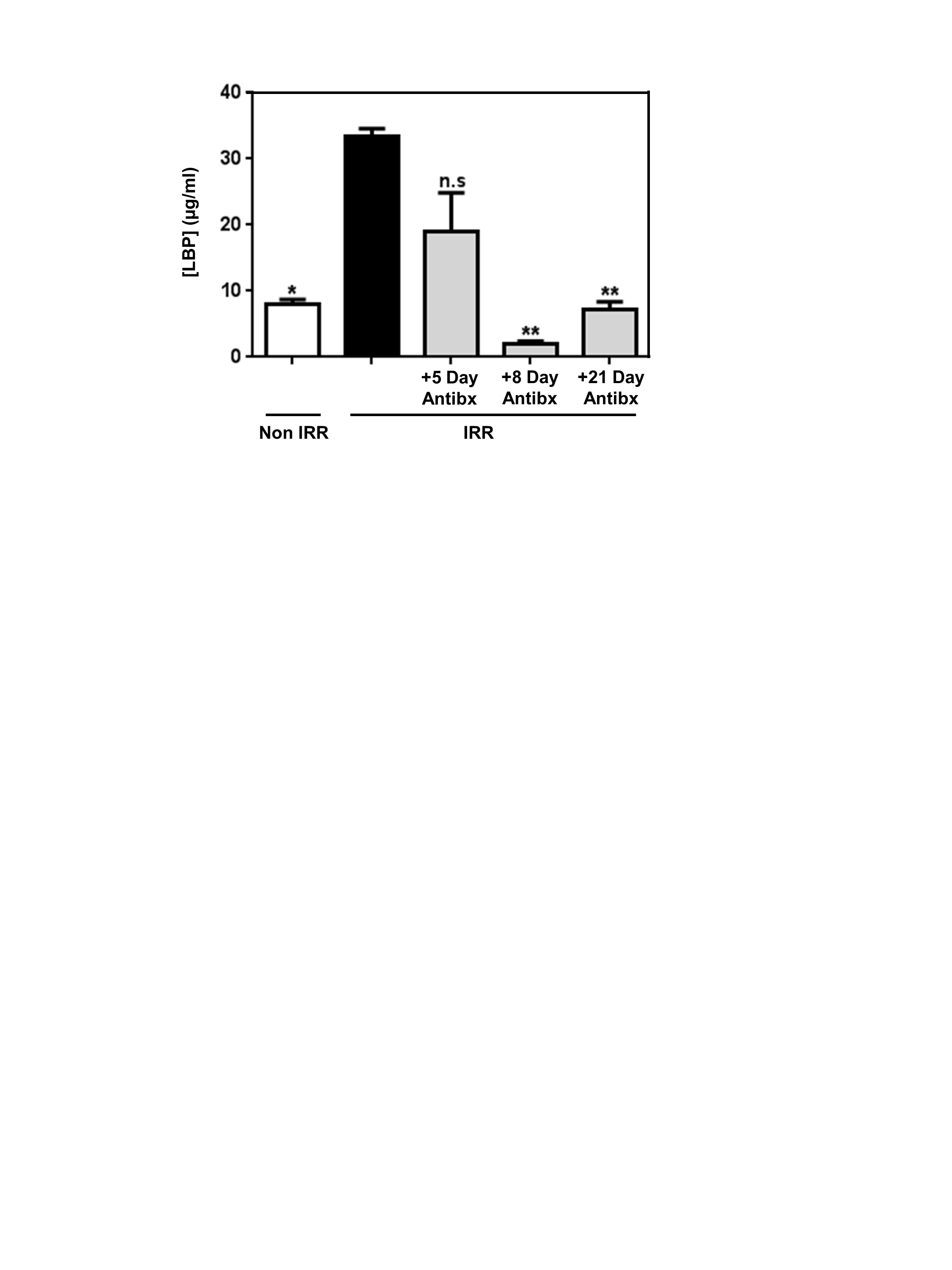

Supplement: S5 Fig — BALB/c mice were treated with antibiotics for different lengths of time, as indicated, before irradiation. Sera were collected 24h after irradiation. Sera from non-irradiated and irradiated mice served as negative and positive controls respectively. Concentration of LBP in serum is presented as means ± SD (n = 4–6) and compared to non-irradiated mice for statistical significance. (TIF) [file pone.0130041.s005.tif]

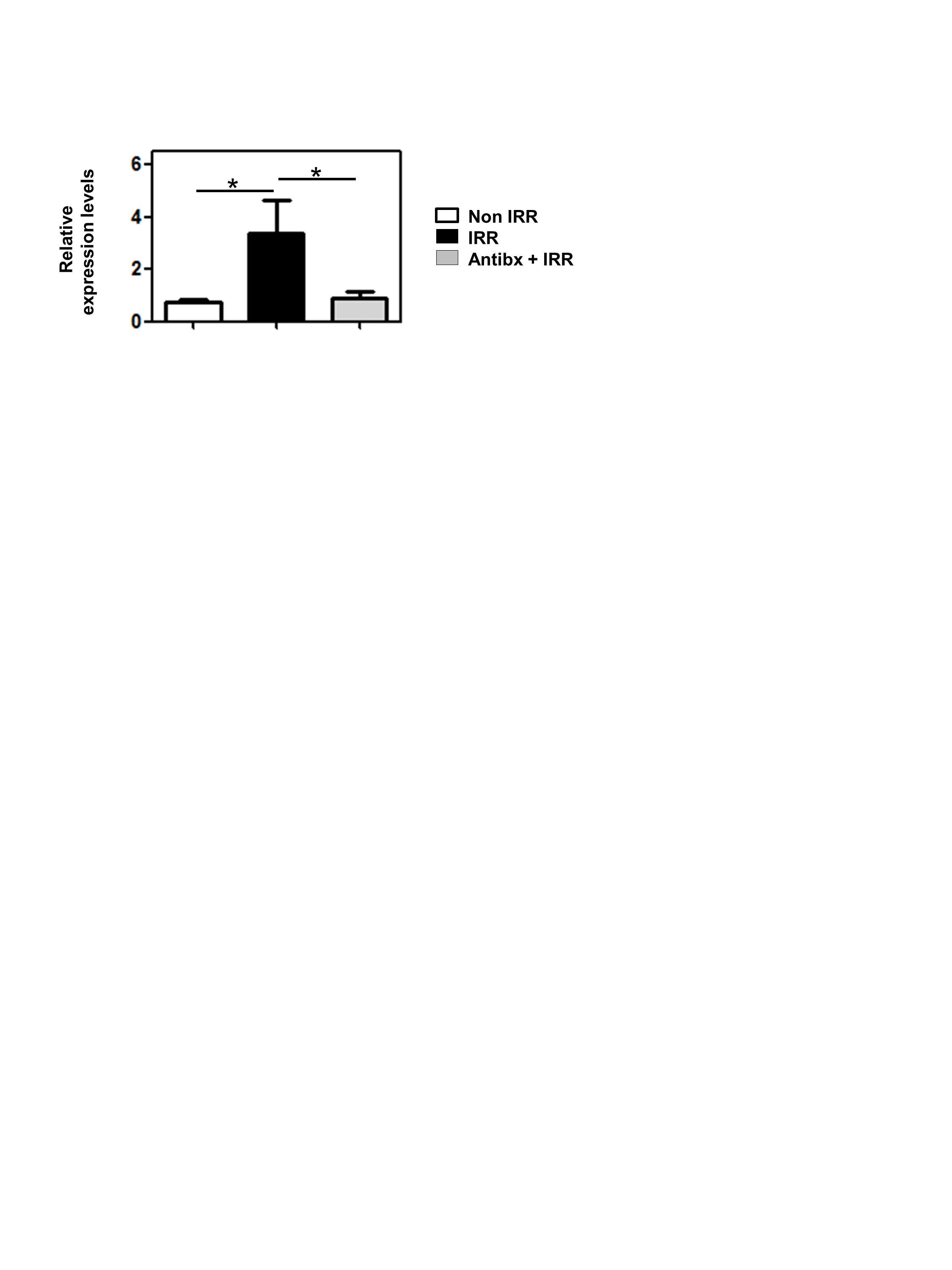

Supplement: S6 Fig — Liver samples from non-treated (Non IRR), irradiated (IRR) and antibiotic-treated irradiated (Antibx + IRR) BALB/c mice were collected 24 h after irradiation and immerged in 5 volumes of RNAlater solution (Ambion). Total RNA extraction was performed using QIAzol Lysis Reagent (Qiagen) and the RNA samples were treated with RQ1 RNase-Free DNase (Promega) to remove genomic DNA contamination. cDNA was synthetized from 500 ng of RNA using the high-capacity cDNA reverse transcription kit (Applied Biosystem). Real-time qPCR was performed using TaqMan specific primers (SOCS1 and Gapdh I.D. of Mm00782550_s1 and Mm99999915_g1 respectively) and TaqMan Universal PCR Master Mix (Applied Biosystem). SOCS1 mRNA relative expression levels are represented as mean ± SD (n = 3–5). Experiment was performed three times. (TIF) [file pone.0130041.s006.tif]

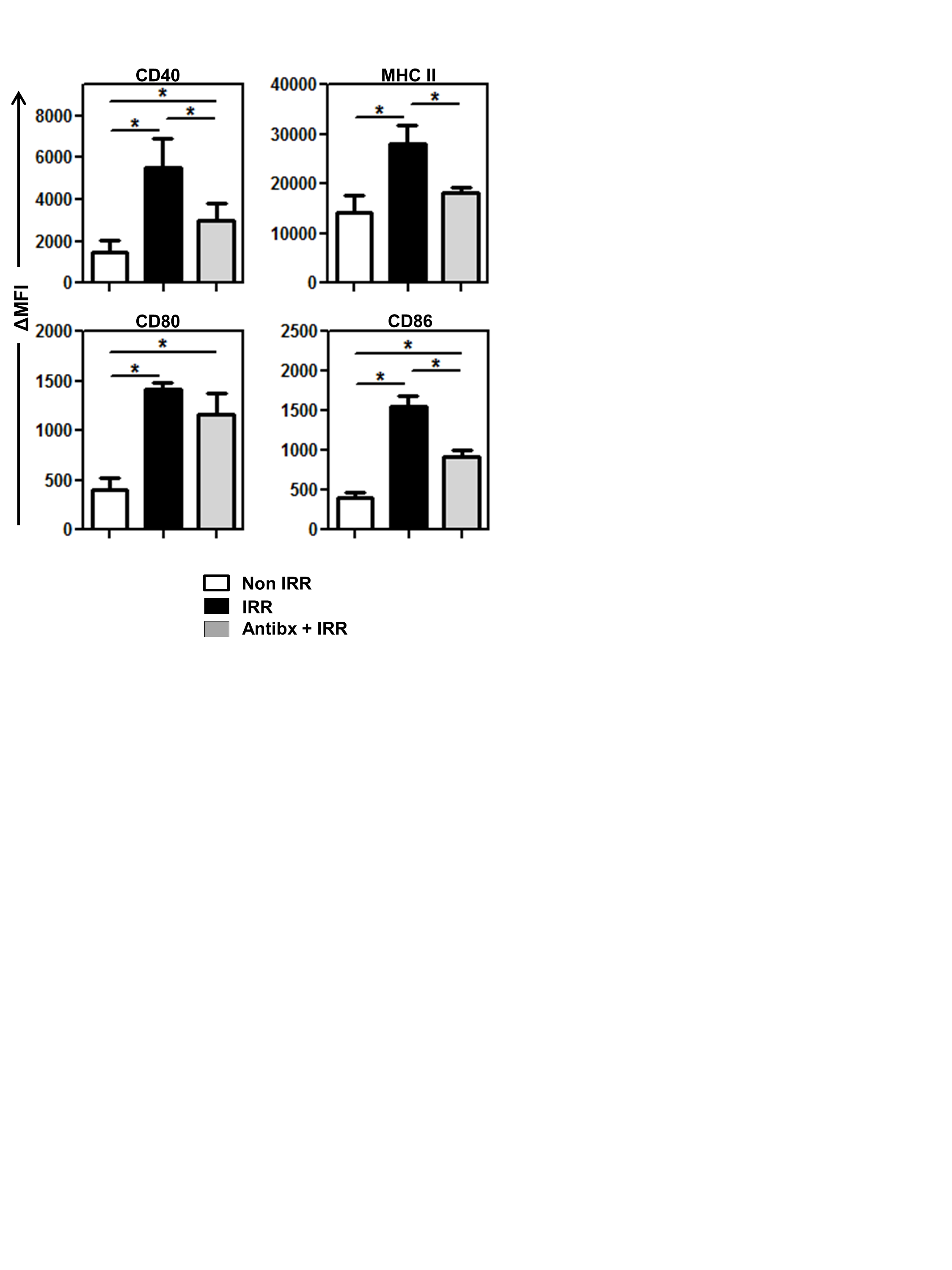

Supplement: S7 Fig — Non-irradiated, irradiated and antibiotic-treated irradiated groups of BALB/c mice have been described in Fig 2. Mice were sacrificed 24h after irradiation and the expression of CD40, MHC class II, CD80 and CD86 on gated living CD3- CD19- DX5- CD11c+ MHC II+ DC from the mesenteric LN was analyzed by FACS. Increase in MFI respect to isotype-matched controls is represented as means ± SD (n = 3–4) from one representative experiment out of three. (TIF) [file pone.0130041.s007.tif]

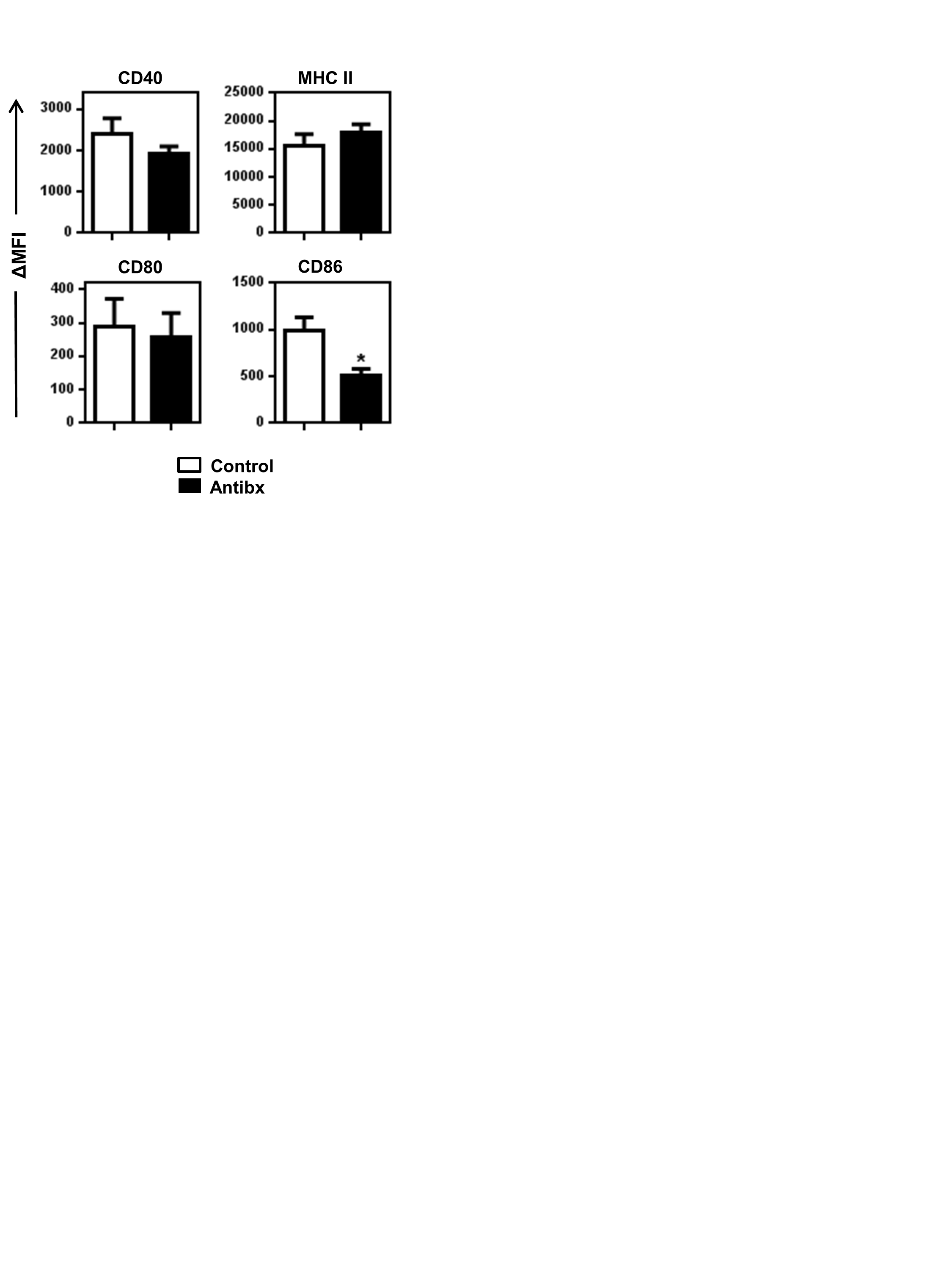

Supplement: S8 Fig — Antibiotic-treated BALB/c mice and non-treated controls were sacrificed 8 days after starting treatment. Expression of CD40, MHC class II, CD80 and CD86 on gated living CD3- CD19- DX5- CD11c+ MHC II+ DC was analyzed by FACS. Increase in MFI respect to isotype-matched controls is represented as means ± SD (n = 3–4) from one out two independent experiments. (TIF) [file pone.0130041.s008.tif]
